# Participatory and Receptive Arts Engagement in Older Adults: Associations with Cognition Over a Seven-Year Period

**DOI:** 10.1080/10400419.2023.2247241

**Published:** 2023-08-29

**Authors:** Jessica K. Bone, Daisy Fancourt, Jill K. Sonke, Feifei Bu

**Affiliations:** a University College London; b University of Florida

## Abstract

There is growing evidence for the impact of arts engagement on later life cognition. However, confounding by socioeconomic factors may have led to an overestimation of this association. We analyzed data from 4,344 older adults in the Wisconsin Longitudinal Study. We measured participatory (e.g. painting, making music, crafts) and receptive (e.g. concert, play, museum) arts engagement separately. Participants completed six neurocognitive tests measuring two distinct domains of cognitive function (episodic/working memory and executive function/language) concurrently and seven years later. We used inverse probability of treatment weighting (IPTW) to remove confounding by a range of demographic and socioeconomic factors. Engaging in participatory or receptive arts for up to one hour per week (but not more frequently) was associated with better subsequent executive function/language. Similarly, engaging in receptive arts activities for up to three hours per week (but not more frequently) was associated with better subsequent episodic/working memory. These effects were of similar sizes to doing vigorous physical activity for up to one hour per week. However, our findings also highlight key methodological issues when exploring the relationship between arts engagement and cognition that should be considered in future studies, including measurement bias, life-course stage, length of follow-up, variation in outcomes, attrition, and missing data.

## Introduction

The United States (US) population is aging at an increased pace. By 2060, adults aged 65 and above are projected to form 23% of the US population, rising from the 16% they comprise now (Vespa, Medina, & Armstrong, 2020). Among this aging population, cognitive decline becomes an increasingly important issue. Worsening memory (ability to hold, manipulate, and recall information), executive function (EF; skills needed to control behavior), capacity for learning, and other cognitive abilities are associated with lower quality of life, a lack of functional independence, and the onset of dementia (Buckley et al., 2015; Gaugler, Duval, Anderson, & Kane, 2007; Jekel et al., 2015). In the US, it is estimated that 22% of adults aged over 70 have cognitive impairment, and the risk of cognitive decline may be increasing over time (Hale, Schneider, Gampe, Mehta, & Myrskylä, 2020; Plassman et al., 2011). Cognitive decline is therefore likely to aggravate existing strains on health and social care systems. Identifying strategies to prevent or delay cognitive decline has been recognized as a public health priority (Shah et al., 2016; World Health Organization, 2012). One potential strategy, which has been receiving increasing attention, is engagement in the arts.

Artistic and cultural activities involve complex cognitive tasks and may enable individuals to lead a more active and socially engaged life that is neuroprotective (Christie et al., 2017; Fratiglioni, Paillard-Borg, & Winblad, 2004). For example, arts activities involve challenging and stimulating experiences, which may enhance neuronal structure and brain function, and thus contribute to increased cognitive reserve (Clare et al., 2017). Arts activities such as making music recruit bilateral temporal, frontal, and parietal neural circuits, which also underlie cognitive processes such as memory, EF, and language (Janata, Tillmann, & Bharucha, 2002). The “use it or lose it” hypothesis suggests that intellectually stimulating activities are needed in everyday life to prevent deterioration in cognitive function (Hultsch, Hertzog, Small, & Dixon, 1999). Additionally, arts engagement is associated with increases in positive affect and other aspects of wellbeing in older adults (Bone et al., 2021), which have been linked with a lower risk of cognitive decline (Allerhand, Gale, & Deary, 2014).

With these mechanisms in mind, the impact of arts and cultural activities on cognition has been investigated in large longitudinal population-based studies in various regions of the world. For example, more frequent receptive cultural engagement (visiting museums, galleries, and exhibitions and going to the theater, concerts, and opera) has been associated with better EF and memory ten years later and lower risk of dementia over the subsequent 12 years (Fancourt & Steptoe, 2018; Fancourt, Steptoe, & Cadar, 2018, 2020; Petrovsky, Wu, Hodgson, & Dong, 2021; Rajan, Rajan, Manning, & Evans, 2018). Moreover, reading books, dancing, and other creative participatory activities (e.g., painting, sewing, playing music) have been linked to increased intellectual functioning, reduced cognitive decline (including global cognition, language, and EF), and lowered incidence of dementia and Alzheimer’s disease over periods of up to 20 years (Bavishi, Slade, & Levy, 2016; Iwasa et al., 2012; Schooler & Mulatu, 2001; Sugita, Ling, Tsuji, Kondo, & Kawachi, 2021; Verghese et al., 2003; Wang et al., 2013; Wang, Karp, Bengt, & Laura, 2002). Reviews of a wide range of intervention studies also demonstrate that participatory arts interventions, such as music training, dance, expressive writing, theater, and visual arts, may lead to improvements in memory, problem solving, and EF (Christie et al., 2017; Noice, Noice, & Kramer, 2014; Tomporowski & Pesce, 2019; Whitty et al., 2020).

However, there are several limitations to existing evidence. Many studies have included a range of artistic, creative, and cultural activities, grouped them together, and explored overall associations with cognition (e.g., Eriksson Sörman, Sundström, Rönnlund, Adolfsson, & Nilsson, 2014; Schooler & Mulatu, 2001; Wang et al., 2013; Wang, Karp, Bengt, & Laura, 2002). This ignores the distinction between activities that are receptive, experiencing arts as an audience member without active involvement, and those that are participatory, requiring the creation of, and active participation, in the arts (Fancourt & Finn, 2019; Fancourt, Aughterson, Finn, Walker, & Steptoe, 2021; Tymoszuk et al., 2021).

Park, Gutchess, Meade, and Stine-Morrow (2007) proposed that any behavior may be receptive (using existing skills and schema) or productive (acquiring new skills and schema), and it is primarily productive engagement that will affect cognitive function as it is more likely to stimulate and develop new neural pathways. These classifications may not be interchangeable with receptive and participatory arts engagement, and specific activities might be either receptive or productive (e.g., singing a familiar song versus learning new songs). However, participatory arts are much more likely to involve productive behavior than receptive arts. According to this hypothesis, we would expect participatory arts to be more beneficial for older adults’ cognition than receptive arts. Yet, there is very little evidence comparing the two forms of engagement. Some small (*n* = 28 to *n* = 221) intervention studies have provided preliminary evidence that productive arts interventions (e.g., quilting, digital photography, or theater arts) are more beneficial for memory, problem solving, and functional connectivity in the brain than receptive arts interventions (e.g., art evaluation, viewing visual art; Bolwerk et al., 2014; Noice, Noice, & Staines, 2004; Park et al., 2014). To our knowledge, these findings have not yet been replicated in population-based studies with longer follow-up periods and larger, more diverse, samples.

It also remains unclear whether any positive impacts of arts engagement on cognitive decline are independent of broader social, structural, and health-related determinants. Many factors that are related to later life cognition, such as age, gender, race/ethnicity, socioeconomic position, educational attainment, chronic disease, risk factors for vascular disease, and sleep are also likely to influence frequency of arts engagement and structural barriers to engaging in the arts (Bone et al., 2021; Dominguez et al., 2021; Fluharty et al., 2021; Koster et al., 2005; WHO guidelines, 2019; Wilson et al., 2009). Socioeconomic factors have been shown to explain much of the association between receptive cultural engagement and cognition (Fancourt & Steptoe, 2018). Although population-based studies have adjusted for these sociodemographic factors, residual imbalance between those who do and do not engage in the arts can still bias results (Shah, Laupacis, Hux, & Austin, 2005). Some intervention studies have randomized participants to overcome confounding, but they generally only have short follow-up periods and have included small samples that are prone to selection bias and may result in unsuccessful randomization, meaning causal inferences cannot be made without measuring all covariates (Bolwerk et al., 2014; Noice, Noice, & Staines, 2004; Park et al., 2014).

Therefore, in this study, we tested the associations between frequency of participatory and receptive arts engagement and cognition seven years later in older adults. Participants were aged 63 to 72 years, so of an age at which cognitive function often starts to decline (Zaninotto, Batty, Allerhand, & Deary, 2018). Participatory arts activities included painting, drawing, playing a musical instrument, and doing crafts or hobbies. Receptive arts included going to a lecture, concert, play, museum, movie, or other similar activity. We used data from the Wisconsin Longitudinal Study, a longitudinal cohort study of older adults in the US (Herd, Carr, & Roan, 2014). To address the issue of confounding by demographic and socioeconomic factors, we analyzed data using inverse probability of treatment weighting (IPTW), also known as propensity score weighting. We hypothesized that more frequent arts engagement, particularly in participatory activities, would be associated with better cognition seven years later (measured using two domains: memory and EF/language). Additionally, to assess the clinical significance of these associations, we compared the effects of arts engagement on cognition to the effects of vigorous physical activity, which has well-established benefits for later life cognition (Baumgart et al., 2015; Gomez-Pinilla & Hillman, 2013; Lista & Sorrentino, 2010).

## Methods

### Sample

Participants were drawn from the Wisconsin Longitudinal Study (WLS), which has followed a random sample of one third of the students graduating from Wisconsin high schools in 1957 (*n* = 10,317; Herd, Carr, & Roan, 2014). Survey administration occurred around ages 17–18 (1957), 35–36 (1975; response rate 90%), 53–54 (1993; response rate 87%), 64–65 (2004; response rate 86%), and 71–72 (2011; response rate 74%). The sample is representative of high school graduates in 1957 living in Wisconsin. As reflected in the WLS data, very few graduates were of ethnicities other than White in 1957. We therefore could not report or adjust for race/ethnicity (<0.5% of participants were of ethnicities other than White; Herd, Carr, & Roan, 2014). This sample is thus not representative of the US population today (US Census Bureau, 2021), preventing broader population inferences. However, WLS has data on family background, educational and employment experiences, socioeconomic position, adolescent characteristics, social engagement, physical and mental health, psychological wellbeing, cognition, and mortality. Surveys have been completed in-person, by mail, and by telephone (Herd, Carr, & Roan, 2014).

**Figure 1.**
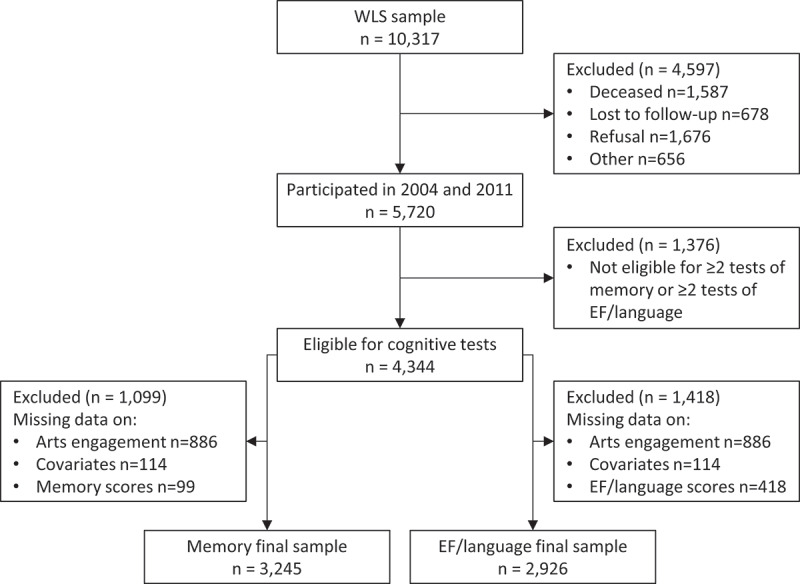
Flow diagram showing the number of participants eligible for and included in the two analytical samples.

In WLS, measures of cognition were administered in 2004 and 2011. Hence, in this study, we used data collected in 2004 (which we refer to as baseline) and 2011 (follow-up), when participants were mainly aged 64–65 and 71–72 (Hauser, Sewell, & Herd, 2021). Figure 1 shows the number of participants eligible for and included in our study. From the original sample of 10,317, we excluded those who did not participate in 2004 or 2011. Of the remaining 5,720 participants, we included those who were eligible for enough cognition measures in 2004 and 2011. In both years, participants were randomly selected to complete a total of up to six cognitive tests, which we grouped as measuring episodic/working memory (immediate recall, delayed recall, and digit ordering tests) or EF/language (similarities, letter fluency, and category fluency tests; see cognition section). Given that not all participants were randomly selected for all cognitive tests in each year, we included participants if they were eligible for at least two of the three tests of memory or EF/language. Therefore, participants had to be eligible for two out of the immediate recall, delayed recall, and digit ordering tests, or two out of the similarities, letter fluency, and category fluency tests in both 2004 and 2011. As different participants were eligible for sufficient tests of memory and EF/language, we included separate analytical samples for each outcome.

For our main analyses, we limited our two analytical samples to participants with no missing data on arts engagement, covariates, and sufficient cognitive tests at baseline and follow-up. The proportion of missing data ranged from 0 to 18% across variables (Table S8), with 18% of participants missing data on participatory arts engagement and 16% missing data on receptive engagement. Excluding those with missing data resulted in a final analytical sample of 3,245 participants for memory and 2,926 for EF/language.

### Participatory arts engagement

As participatory and receptive arts activities differ in activity content and context (Fancourt & Finn, 2019; Fancourt, Aughterson, Finn, Walker, & Steptoe, 2021; Tymoszuk et al., 2021), and may have differing effects on cognition, we planned a priori to measure time spent on these activity types separately. In the 2004 mail survey, participants completed a questionnaire on social and civic participation, which measured engagement in a total of 25 activities. Three items measured participatory arts engagement: during the past year, how many hours per month did you 1) paint, draw, or do another form of art; 2) play a musical instrument; 3) do crafts or hobbies such as needlework, woodworking, model trains, jigsaw puzzles, etc. We calculated the total time spent on participatory arts in the last month by summing responses to these questions. Given that effects may not be linear and this variable had a large range and was positively skewed, we categorized total scores into quartiles: no engagement (0 hours), low engagement (1–4 hours per month), moderate engagement (5–12 hours per month), and high engagement (13 hours or more per month). Low engagement was equivalent to between fifteen minutes and one hour per week, moderate one to three hours per week, and high more than three hours per week.

### Receptive arts engagement

We combined responses to two questions from the social and civic participation questionnaire as indicators of receptive arts engagement: during the past year, how many hours per month did you spend going to 1) the movies; 2) a lecture, concert, play, museum, or other similar activity. To enable comparison to participatory arts, total scores were categorized in the same way: no engagement (0 hours), low engagement (1–4 hours), moderate engagement (5–12 hours), and high engagement (13 hours or more).

### Physical activity

We included physical activity as a positive control in sensitivity analyses. We combined responses to two items from the social and civic participation questionnaire as indicators of physical activity: during the past year, how many hours per month did you do 1) vigorous physical activities that you do alone, such as jogging, swimming, biking, or going to the gym by yourself; 2) vigorous physical activities that you do with others such as playing team sports, jogging, swimming, biking, or going to the gym with friends. To enable comparison to arts engagement, total scores were split to represent: no engagement (0 hours), low engagement (1–4 hours), moderate engagement (5–12 hours), and high engagement (13 hours or more). The World Health Organization recommends that adults should engage in at least 1.25 to 2.5 hours of vigorous-intensity physical activity per week (i.e., 5–11 hours per month; World Health Organization, 2018), which is equivalent to the moderate engagement category.

### Cognition

The six cognitive tests in WLS were completed over the phone in 2004 and in person in 2011. These were: 1) a similarities test from a subscale of the Wechsler Adult Intelligence Scale (WAIS) Revised (abstract reasoning; Wechsler, 1981); 2) a letter fluency task (phonemic verbal fluency; Tombaugh, Kozak, & Rees, 1999); 3) a category fluency task (semantic verbal fluency; Tombaugh, Kozak, & Rees, 1999); 4) a digit ordering test adapted from the WAIS-III (working memory; Wechsler, 1997); and 5–6) immediate and delayed word recall tests from the Telephone Interview for Cognitive Status (episodic memory; Brandt, Spencer, & Folstein, 1988). All participants were eligible to complete the similarities test in both years and the letter fluency task in 2011. A random 80% subsample of participants were eligible to complete the letter fluency task in 2004, and the digit ordering and recall tests in 2004 and 2011 (with different subsamples taken in each year). A different random 50% subsample of participants were eligible to complete the category fluency task in 2004 and 2011.

In line with previous studies that have performed factor analyses of scores on these tests in 2004 and 2011 (Greenfield & Moorman, 2019, 2019; Greenfield, Moorman, & Rieger, 2021; Moorman, Carr, & Greenfield, 2018), we used a two-factor solution of 1) memory and 2) EF/language. As outlined above, memory included scores on the tests of immediate recall, delayed recall, and digit ordering, so provided a broad measure of both episodic and working memory, gaging participants’ ability to remember information in the short-term and after some time has elapsed as well as their ability to hold and manipulate information. EF/language included scores on the tests of similarities, letter fluency, and category fluency, which measured participants’ ability to identify what different objects might have in common and think of as many words starting with a letter or belonging to a category as they can, thus assessing some of the cognitive skills needed to control behavior. As the six tests were scored on different scales, we calculated the percent of maximum possible scores for each test (Cohen, Cohen, Aiken, & West, 1999). For each outcome (memory vs EF/language), we created summary scores as the average of scores on the three tests. As we included participants missing scores on up to one test for each outcome, their summary scores were created by averaging scores on the other two tests. We then standardized memory and EF/language scores within our analytical sample (mean = 0, standard deviation = 1). The standardized score thus represents the number of standard deviations each participant’s raw score is from the overall mean of that measure.

### Covariates

We included a range of demographic and socioeconomic factors measured at baseline (2004). These were gender (men, women), age (63–64, 65–67 years), marital status (married, unmarried [separated/divorced/widowed/never married]), highest level of education (high school or less, some college, undergraduate degree, postgraduate degree), employment status (as reported by WLS; employed, unemployed, retired), and household income (quartiles: $0–$36,000, $36,001-$57,000, $57,001-$91,000, $91,001-$710,000). Given the evidence that childhood socioeconomic position is associated with both later life cognition (Greenfield, Moorman, & Rieger, 2021) and arts engagement (Bone et al., 2021), we also included the head of household’s level of education in 1957 as an indicator of childhood socioeconomic position. We will refer to this as parental education (high school or less, college or above).

### Statistical analysis

To address the issue that there are a range of structural determinants of arts engagement, we used inverse probability of treatment weighting (IPTW). This approach, also referred to as propensity score weighting (Guo & Fraser, 2015; Imbens, 2000), creates a pseudo-population in which the treatment (arts engagement) no longer depends on the covariates, and the outcome (cognition) is conditionally independent of the treatment. Confounding by all observed covariates is thus removed. In this way, IPTW simulates a trial with the measured covariates randomized between groups. We estimated the difference between the outcome if the entire sample participated in each level of arts engagement (low, moderate, high) and the outcome if the whole sample did not engage in the arts (the average treatment effect; ATE). This provides the average effect of each level of arts engagement on subsequent cognition.

To perform IPTW, we estimated a propensity score for each participant, indicating how likely they were to be in each category of arts engagement at baseline. Propensity scores were calculated from a multinomial logit model including all demographic and socioeconomic covariates and the baseline cognition measures. The inverse of the propensity score was then used as a sampling weight, with each participant’s weight equal to the inverse of the probability of receiving the treatment that they received (i.e., no, low, moderate, or high engagement). With this sampling weight, we could test whether arts engagement at baseline was associated with cognition at follow-up independent of covariates and baseline cognition. Including baseline cognition meant our analyses tested whether arts engagement was associated with change in cognition seven years later. We did this separately for participatory and receptive arts engagement and each outcome. We report the balance of covariates over treatment groups before and after weighting (Figure S1). All analyses were performed using Stata 17 (StataCorp, 2021).

### Sensitivity analyses

We performed a series of sensitivity analyses. First, we compared the effects of arts engagement on cognition to the effects of physical activity. Physical activity can be interpreted as a positive control, as it has well-established benefits for later life cognition (Gomez-Pinilla & Hillman, 2013; Lista & Sorrentino, 2010). This allowed us to compare the sizes of the effects of physical activity and arts engagement on cognition.

Second, we excluded attending the movies from our measure of receptive arts engagement because previous research found that, although other cultural activities were beneficial for cognition, going to the cinema was not (Fancourt & Steptoe, 2018). We then replicated the main analyses using this measure of receptive arts engagement (going to a lecture, concert, play, museum, or other similar activity). We also separately examined whether attending the movies was associated with cognition.

Third, given that propensity score methods are more frequently used for treatments that are binary (i.e., participants either did or did not receive a treatment) than for treatments with multiple values, we performed a sensitivity analysis using binary indicators of participatory and receptive arts engagement. We compared any engagement in participatory or receptive arts (≥1 hour per month) to no engagement (0 hours per month)

Fourth, we cross-validated our findings using an alternative approach; linear regression models tested whether the two types of art engagement (participatory and receptive) were associated with the two cognitive outcomes (memory and EF/language). We adjusted all models for baseline cognition. Adjusting longitudinal models for cognition measured at baseline considers that cognition at follow-up is not only related to arts engagement, but also to previous cognition. The fully adjusted model thus estimates the association between arts engagement and change in cognition seven years later. Models are presented before and after adjustment for covariates.

### Multiple imputation

Given concerns about biases due to limiting our sample to complete cases, we performed another series of sensitivity analyses using multiple imputation. Limiting the sample to those eligible for sufficient cognitive tests in 2004 and 2011 resulted in a total of 4,344 participants for imputation (see Table S2 for a comparison of participant characteristics). Following guidance on how best to implement IPTW to avoid bias and loss of precision when data are missing, we combined multiple imputation with IPTW (Granger, Sergeant, & Lunt, 2019). For participants with missing data on any variable (memory, EF/language, arts engagement, or covariates), we imputed data using multiple imputation by chained equations (MICE; White, Royston, & Wood, 2011). We used truncated linear, logistic, and ordinal regression according to variable type, generating 20 imputed data sets. The imputation model included all variables used in analyses and general health status as an auxiliary variable. After checking model convergence, we first assessed the imputed data numerically, making external checks that values were plausible. We then performed internal checks, tabulating summary statistics and using plots to assess for discrepancies between the observed and imputed data, ensuring the distribution of imputed data were similar to the observed data (Nguyen, Carlin, & Lee, 2017). All variables were successfully imputed.

After imputation, we performed IPTW using two approaches as there has been debate over which is best (Granger, Sergeant, & Lunt, 2019). Firstly, we used the *“teffects ipw”* Stata command in combination with “*mi estimate”* to fit the IPTW estimation command to our multiply imputed data. We refer to this as the *“conventional approach”*. Secondly, we used IPTW individually in each imputation to obtain 20 effect estimates. These estimates were then combined using Rubin’s rules to produce estimates of overall exposure effects, standard errors (comprised of both the between-imputation and within-imputation variance), and confidence intervals. This is known as the “*within approach*” and has been shown to produce unbiased estimates, particularly in comparison with other approaches, which may produce biased estimates and unrealistic confidence intervals (Granger, Sergeant, & Lunt, 2019).

## Results

**Figure 2.**
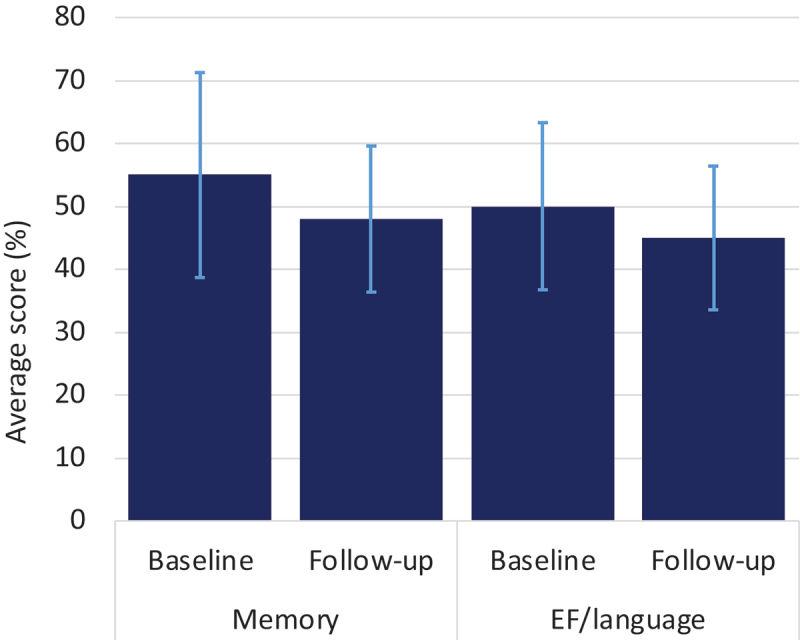
Average raw cognition scores across the study period. Standard deviations are shown with light blue error bars. Total possible scores range from 0 to 100, as they are average of the percent of maximum possible scores on the three relevant tests (memory: immediate recall, delayed recall, digit ordering; EF/language: similarities, letter fluency, category fluency).

Participants were aged 63 to 67 years at baseline and 70 to 74 at follow-up. Before IPTW, 47% were male, 79% were married, 46–47% were employed, and 43% were retired at baseline (Table 1). Performance on the memory and EF/language cognitive tasks worsened slightly in the seven years from baseline to follow-up (Figure 2). At baseline, the raw mean memory score was 55% (standard deviation [SD] = 16, range 0–100%) and EF/language was 50% (SD = 13, range 15–90%), which declined to 48% (SD = 12, range 0–100%) and 45% (SD = 11, range 6–85%) respectively at follow-up. Within-individual variation accounted for approximately 51% of the overall variation in memory and 69% in EF/language.

**Table 1.** Characteristics of the samples for each outcome at baseline.

	Memory (*N* = 3,245)	EF/language (*N* = 2,926)
	Proportion	
**Participatory arts engagement**		
None	39%	38%
Low	21%	21%
Moderate	18%	19%
High	22%	22%
**Receptive arts engagement**		
None	29%	29%
Low	41%	41%
Moderate	24%	24%
High	6%	6%
**Age**		
63–64 years	67%	68%
65–67 years	33%	32%
**Gender**		
Men	47%	47%
Women	53%	53%
**Marital status**		
Married	79%	79%
Unmarried	21%	21%
**Highest education**		
High school or less	52%	52%
Some college	15%	15%
Undergraduate	15%	15%
Postgraduate	18%	18%
**Employment status**		
Employed	47%	46%
Unemployed	10%	11%
Retired	43%	43%
**Household income**		
$0–$36,000	20%	20%
$36,001–$57,000	27%	27%
$57,001–$91,000	26%	26%
≥$91,001	27%	27%
**Parental education**		
High school or less	85%	85%
College or above	15%	15%

Notes: Not all participants were randomly selected for all cognitive tests in each year, so different subsamples who completed sufficient tests of memory and executive function (EF)/language were included. Low engagement is 1–4 hours per month, moderate engagement 5–12 hours, and high engagement ≥13 hours per month.

Overall, 21% of participants reported engaging in participatory arts activities (painting, drawing, playing a musical instrument, arts, crafts, or hobbies) at low frequencies, 18–19% at moderate, and 22% at high frequencies (Table 1). Low engagement was equivalent to between one and four hours per month, moderate five to 12 hours per month, and high 13 hours or more per month. In contrast, 41% of participants reported engaging in receptive arts activities (lecture, concert, play, museum, or movies) at low frequencies, 24% at moderate, and 6% at high frequencies.

Looking across activity types, 14% of participants did not report either participatory or receptive arts engagement, 15% did only participatory arts, 24% did only receptive arts, and 47% did both participatory and receptive arts activities in the last year. However, frequency of engagement in activity types differed. For example, only 2% of the sample engaged in both participatory and receptive activities at high frequencies.

## Balance of covariates

We used standardized differences to compare the balance in measured baseline covariates between participants at each level of arts engagement (Austin & Stuart, 2015). Before weighting, there were differences across all covariates according to the level of engagement in arts activities (Figure S1). Standardized differences were large, ranging from 0.00 to 0.56 (Table S1). Using IPTW corrected the balance of covariates across these groups, with standardized differences greatly reduced and below the threshold for meaningful imbalance in covariates (0.10; Austin & Stuart, 2015) for all except two of the 39 comparisons (income quartile 2 in receptive arts models). Comparing the distributions of covariates between groups, the variances were also more similar after IPTW (variance ratios closer to 1; Table S1). IPTW therefore sufficiently removed residual systematic differences in observed baseline characteristics between groups.

## Memory

We found no evidence that engagement in participatory arts was associated with memory seven years later (Table 2). In contrast, more receptive arts engagement was associated with better memory. Engaging in receptive activities at a low frequency was associated with a 0.09 (95% CI = 0.01 to 0.17) standard deviation higher memory score seven years later compared to no engagement. Moderate frequency engagement was associated with a 0.11 (95% CI = 0.01 to 0.20) standard deviation higher memory score versus no engagement. However, high frequency engagement was not associated with subsequent memory.

**Table 2.** Associations between arts engagement and the two cognition outcomes using inverse probability of treatment weighting.

	Memory (*N* = 3,245)				EF/language (*N* = 2,926)			
	Participatory arts		Receptive arts		Participatory arts		Receptive arts	
	ATE	95% CI	ATE	95% CI	ATE	95% CI	ATE	95% CI
None	-	-	-	-	-	-	-	-
Low	0.03	−0.05 to 0.12	**0.09**	**0.01 to 0.17**	**0.08**	**0.001 to 0.15**	**0.10**	**0.02 to 0.17**
Moderate	0.08	−0.01 to 0.17	**0.11**	**0.01 to 0.20**	0.07	−0.01 to 0.15	0.02	−0.08 to 0.11
High	−0.01	−0.10 to 0.08	−0.02	−0.17 to 0.13	0.03	−0.05 to 0.12	0.07	−0.08 to 0.22

Notes: ATE: average treatment effect. CI: confidence intervals. For both participatory and receptive arts engagement, the control (reference) group was no engagement. Memory and EF/language were standardized, so ATEs are in standard deviation units. Low engagement is 1–4 hours per month, moderate engagement 5–12 hours, and high engagement ≥13 hours per month. Bold text indicates 95% CIs do not include 0.

### Executive function/language

There was some evidence that engagement in both participatory and receptive arts was associated with subsequent EF/language (Table 2). Compared to no engagement, low frequency participatory engagement was associated with a 0.08 (95% CI = 0.00 to 0.15) standard deviation higher EF/language score seven years later. Similarly, low frequency receptive engagement was associated with a 0.10 (95% CI = 0.02 to 0.17) standard deviation higher EF/language score seven years later versus no engagement. However, for both activity types, neither moderate nor high frequency engagement were associated with EF/language seven years later.

### Sensitivity analyses

#### Physical activity

In our first sensitivity analysis, we estimated the effects of physical activity on cognition. Overall, 10% of participants reported engaging in physical activity at low frequencies, 16% at moderate, and 21% at high frequencies. Of the participants who did not engage in any type of arts, 73% also did not do physical activity. Of those who did engage in the arts, 49% also reported doing physical activity and 51% did not. The effects of physical activity on cognition were similar to those of participatory and receptive arts engagement (Table S3). Compared to no physical activity, low frequency engagement was associated with a 0.13 (95% CI = 0.03 to 0.24) standard deviation higher memory score seven years later. For EF/language, both low (ATE = 0.12, 95% CI = 0.03 to 0.22) and high (ATE = 0.08, 95% CI = 0.01 to 0.16) frequencies of physical activity were associated with higher EF/language scores seven years later compared to no engagement.

#### Inclusion of movies in receptive arts engagement

In our second sensitivity analysis, we repeated the main analyses after excluding attending the movies from the measure of receptive arts engagement. Although low frequency receptive engagement was still associated with higher memory scores seven years later, there was no longer evidence for an effect of moderate engagement (Table S4). For EF/language score, the results were consistent with the main analysis; only low frequency receptive engagement was associated with higher EF/language score seven years later. We then tested the association between just attending the movies and cognition, excluding other forms of receptive arts engagement. Attending the movies at low frequencies, compared to never, was associated with higher memory scores seven years later (Table S5). However, there was no evidence for associations when attending more frequently or with EF/language.

#### Binary indicators of arts engagement

In our third sensitivity analysis, comparing any engagement in participatory or receptive arts to no engagement did not alter our findings for subsequent memory. Only receptive engagement was associated with better memory seven years later (Table S6). However, only participatory arts engagement was associated with higher EF/language scores seven years later.

#### Regression models

In our fourth sensitivity analysis, cross validating our findings in linear regression models provided very similar evidence to IPTW (Table S7). After adjusting for covariates, participatory engagement was not associated with subsequent memory, but both low and moderate frequency receptive engagement was associated with higher memory scores seven years later. For EF/language, only low frequency engagement in participatory or receptive activities was associated with higher subsequent cognition.

#### Multiple imputation

The characteristics of our imputed sample (*n* = 4,344) were similar to those of our main analytical samples (Table S2). After imputation, both approaches to IPTW corrected the balance of covariates across the different levels of arts engagement (Figure S2). However, these approaches provided inconsistent results.

The *“conventional approach”* to IPTW provided similar evidence to our main analyses (Table S9). Compared to no engagement, moderate frequency participatory arts engagement was associated with higher memory and EF/language scores seven years later. Both low and moderate frequency receptive arts engagement were associated with higher memory and EF/language scores seven years later versus no engagement. Finally, low frequency physical activity was associated with higher memory scores, and all frequencies of physical activity were associated with higher EF/language scores seven years later.

In contrast, after using the *“within approach”* to IPTW, there was no longer evidence for an effect of any exposure on memory or EF/language (Table S10). Although ATEs were similar to the *“conventional approach”* and the complete case analysis, the confidence intervals from the *“within approach”* were much wider. This may be because standard errors in this approach combine both the between-imputation and within-imputation variance (Granger, Sergeant, & Lunt, 2019).

## Discussion

In this study, we tested the relationships between frequency of participatory (e.g., painting, drawing, playing a musical instrument, arts, crafts, or hobbies) and receptive (e.g., lecture, concert, play, museum, or movies) arts engagement and change in cognition seven years later in older adults. After considering the structural determinants of arts engagement by using IPTW, we found some evidence that engagement in participatory or receptive arts activities was associated with memory and EF/language seven years later. Specifically, low and moderate levels of receptive engagement were associated with better memory, and low frequencies of engagement in both participatory and receptive activities were associated with better EF/language. However, effect sizes were small and there was no evidence that more frequent arts engagement was more beneficial for cognition. Our findings were generally robust to a range of sensitivity analyses apart from multiple imputation, which we discuss below. In the same samples, we also found evidence that low frequencies of vigorous physical activity (e.g., jogging, swimming, biking, or going to the gym) were associated with higher memory and EF/language scores seven years later. Yet, as with arts engagement, there was no dose-response relationship; although high levels of physical activity were associated with better subsequent EF/language, this was a smaller effect than for low levels of physical activity.

## Findings in context

Our findings are surprising given evidence from other longitudinal population-based studies that more frequent participatory and receptive arts engagement is associated with better memory, EF, and intellectual functioning, and lower rates of cognitive decline, dementia, and Alzheimer’s disease (Bavishi, Slade, & Levy, 2016; Fancourt & Steptoe, 2018; Fancourt, Steptoe, & Cadar, 2018, 2020; Iwasa et al., 2012; Schooler & Mulatu, 2001; Verghese et al., 2003; Wang et al., 2013; Wang, Karp, Bengt, & Laura, 2002). Whilst our findings do not negate previous positive results, effect sizes were small and a dose-response relationship between arts engagement and cognition was not found in this sample. It is important to consider why this might be.

One possible explanation is that we used a more sophisticated statistical technique than in previous studies to account for demographic and socioeconomic factors, such as age, gender, socioeconomic position, and educational attainment, as well as baseline cognition, which influence both the frequency of arts engagement and later life cognition (Bone et al., 2021; Fluharty et al., 2021; Koster et al., 2005; WHO guidelines, 2019; Wilson et al., 2009). The mixed evidence after accounting for these factors is consistent with previous findings that socioeconomic factors explain much of the association between cultural engagement and cognition (Fancourt & Steptoe, 2018). It is possible that the lack of dose-response relationship was due to the confounding effects of socioeconomic factors being more effectively removed than in previous studies. Consistent with this, there was stronger evidence for associations in our unadjusted regression sensitivity analyses that was attenuated after adjusting for covariates, suggesting that it was due to socioeconomic differences between groups. Therefore, whilst a social gradient in arts engagement and cognition exists, disentangling the effects of the arts on cognition will be challenging. If this gradient can be reduced and more opportunities for arts engagement presented to individuals from lower socioeconomic backgrounds across the life course, there may be more opportunities for tangible effects on later life cognition.

Alternatively, symptoms of cognitive decline (including prodromal symptoms of dementia) can have effects on behaviors years or even decades before these conditions manifest. As such, individuals on course to experience declines in cognition may have already reduced their engagement in arts and cultural activities before older age (Floud et al., 2021). Thus, our findings could support the proposal that early manifestations of cognitive decline reduce participation in arts engagement and physical activity, and participation in these activities has little effect on subsequent cognition (Floud et al., 2021). The likelihood of biases due to this reverse causality is also increased by the relatively short follow-up period in our study, as the effects of prodromal symptoms of dementia may be greater in longitudinal studies with less than ten years of follow-up (Floud et al., 2021).

## Role of study methodology

It is possible that methodological decisions in this study may have influenced our findings. Understanding these methodological issues could be key to uncovering more about the potential relationship between arts engagement and cognition. For example, small changes in cognition over the course of just seven years (a relatively brief period) may not have been detectable. Indeed, although performance on the cognitive tasks declined during the study period, these changes were small, with an average reduction of only 5–7 points on a 100-point scale. Additionally, our sample focused on adults in their 60s and 70s. This is an age at which cognitive function starts to decline (Zaninotto, Batty, Allerhand, & Deary, 2018). Yet, it remains possible that arts engagement has larger effects later in life, when trajectories of cognitive decline are even steeper (Zaninotto, Batty, Allerhand, & Deary, 2018), or over longer periods. Whilst previous studies have demonstrated associations between arts engagement and cognition even after adjusting for baseline cognition, these studies have typically involved longer follow-up periods of up to 20 years and included older adults (e.g., Fancourt & Steptoe, 2018; Fancourt, Steptoe, & Cadar, 2020; Schooler & Mulatu, 2001; Verghese et al., 2003). Analyses that take account of much longer timescales are therefore needed.

There are further potential issues around attrition. Given that people with poor cognition may have been less likely to complete study measures, we imputed missing data in a series of sensitivity analyses. Multiple imputation is more likely to result in unbiased estimates, with higher validity than listwise deletion, and uses all available data, preserving sample size and statistical power (White, Royston, & Wood, 2011). The combination of multiple imputation with propensity score methods is a relatively new field, and there is ongoing debate as to the best approach (see Granger, Sergeant, & Lunt, 2019 for a summary). We therefore compared the findings from two approaches, but these provided inconsistent evidence. One was similar to our main complete case analyses, but the other indicated that none of participatory or receptive arts engagement or physical activity had an effect on subsequent cognition. Another strategy has also been used for propensity score methods in imputed data (the “across approach;” Granger, Sergeant, & Lunt, 2019), which we had intended to compare to the other two approaches in sensitivity analyses. However, it cannot be used when participants are also missing data on exposures or outcomes, as in our study. Given the inconsistencies in our findings, they should be replicated in larger samples with less attrition as well as using improved methods for combining imputation and propensity score weighting when developed.

It is also notable that, similar to the evidence for arts engagement, only some frequencies of vigorous physical activity were associated with change in memory or EF/language in this study. We did not expect low levels of engagement to show the strongest associations with subsequent cognition. We intended to include physical activity as a positive control, given that it has well-established benefits for later life cognition (Baumgart et al., 2015; Gomez-Pinilla & Hillman, 2013; Lista & Sorrentino, 2010). The lack of consistent association between physical activity and cognition could thus indicate that there was insufficient variation in the outcome for effects to be detected. It is also possible that the categorization of engagement affected our findings, as relatively few people reported engaging in any of the activities at high levels. Our analyses may therefore have been underpowered to detect associations between high engagement and subsequent cognition.

Additionally, we measured only arts engagement and physical activity at baseline, without considering continued or consistent engagement or any new engagement over follow-up, which could have led to an underestimation of the effects on cognition. A systematic review concluded that several months of engagement in musical activities might be needed for older adults to receive the maximum benefits (Christie et al., 2017), and observational research has demonstrated that sustained arts engagement has the largest impact on health (Tymoszuk et al., 2019). Nonetheless, visiting museums, galleries, or exhibitions even once a year has been shown to be protective for some aspects of cognition (Fancourt & Steptoe, 2018; Fancourt, Steptoe, & Cadar, 2020).

## Strengths and limitations

This study has a number of strengths. WLS includes a large cohort of older adults in the US. As participants are all similar ages and were recruited into the cohort at the same period, this removes any potential influence of these factors on our findings. The rich data allowed us to include a range of demographic and socioeconomic covariates in IPTW models, which minimized the risk of bias caused by unobserved heterogeneity. We replicated a previous approach to measuring cognition in WLS (Greenfield & Moorman, 2019; Greenfield, Moorman, & Rieger, 2021; Moorman, Carr, & Greenfield, 2018; Moorman, Greenfield, & Garcia, 2019), splitting it into two factors of memory and EF/language, and were able to use identical measures over a period of seven years. We compared participatory and receptive arts engagement, which has not been done previously in population-based studies, and tested physical activity, allowing us to assess the potential clinical significance of associations.

This study also has several limitations. Our exploration of different types of arts engagement was limited to the activities included in the dataset. WLS asked participants to self-report their current engagement in arts activities. While the question focused on engagement in the past year rather than over longer retrospective timescales, this reporting may still have been biased. We conducted several exploratory sensitivity analyses. Although we compared the effects of arts engagement on cognition to physical activity, we were not able to test whether there was an interaction between these exposures, or whether physical activity mediated the association between arts engagement and cognition. Furthermore, IPTW cannot control for any unmeasured factors that may have influenced both arts engagement and cognition, such as health-related factors or participation in other social activities. But, given the range of covariates included in our models, any remaining unobserved heterogeneity should be relatively small. Future studies could include a larger set of covariates in IPTW to confirm our findings. Factors such as race/ethnicity, neighborhood safety, social contact, health status, and difficulties with activities of daily living may be relevant. We were not able to adjust for race/ethnicity because of the small number of participants of ethnicities other than White in WLS. This makes our findings relevant only for people of White race/ethnicity and not generalizable to older adults in the US population. Although WLS is a valuable source of detailed longitudinal data, it started in 1957 and provides a snapshot of the population of Wisconsin at this time. This was a different social and political context, since which attitudes and laws have changed, particularly toward race/ethnicity. Future research must include more diverse samples, particularly those that provide more equitably robust reflections of the US population and collect more detailed data on race/ethnicity. Finally, our findings should be replicated in larger studies with less attrition.

## Conclusion

In this study, after considering the structural determinants of arts engagement and cognition in this sample, we found some evidence that engaging in receptive arts activities for up to three hours per week (but not more frequently) was associated with better memory performance seven years later. Similarly, engaging in participatory or receptive arts activities for up to one hour per week (but not more frequently) was associated with better EF/language performance seven years later. These effects of arts engagement on cognition were small but of comparable sizes to engaging in vigorous physical activity for up to one hour per week. Our findings point to the importance of properly accounting for demographic and socioeconomic factors that may influence both arts engagement and cognition. However, our paper also highlights some of the key methodological challenges in exploring the relationship between arts and cognition. First, it is possible that limitations such as the small changes in cognition over time and measuring arts engagement and physical activity only at one point in time may have influenced our findings. Second, due to issues with attrition and missing data, our findings were not conclusive. Thus, in other studies, decisions made on these issues could have substantial effects on the results presented. This highlights why future research should incorporate an even wider range of demographic, socioeconomic, health-related, and social factors and include older age cohorts, longer follow-ups, and more details on whether arts engagement is sustained over time as well as testing the consistency of results when different approaches to missing data and attrition are used. Understanding and addressing these issues is vital to truly understanding the presence and extent of a relationship between arts engagement and cognition in older age and thus the role that community-based programs could play in supporting healthy aging.

## Supplementary Material

Supplemental Material

## Data Availability

The data that support the findings of this study are openly available from the Wisconsin Longitudinal Study (WLS) website at https://www.ssc.wisc.edu/wlsresearch/data/.
