# Supplementary material for: Participatory and Receptive Arts Engagement in Older Adults: Associations with Cognition Over a Seven-Year Period
Source: Creat Res J. 2023 Aug 29;36(3):436–50. doi: 10.1080/10400419.2023.2247241 (PMC11318508; doi:10.1080/10400419.2023.2247241)
Supplement: Supplemental Material [file HCRJ_A_2247241_SM2109.docx]

**Supplementary materials**


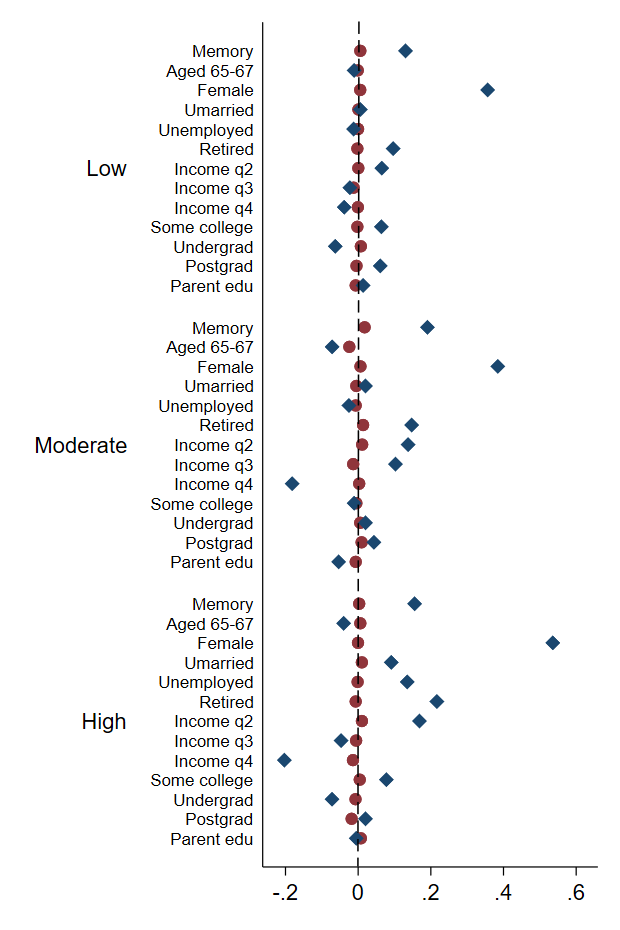

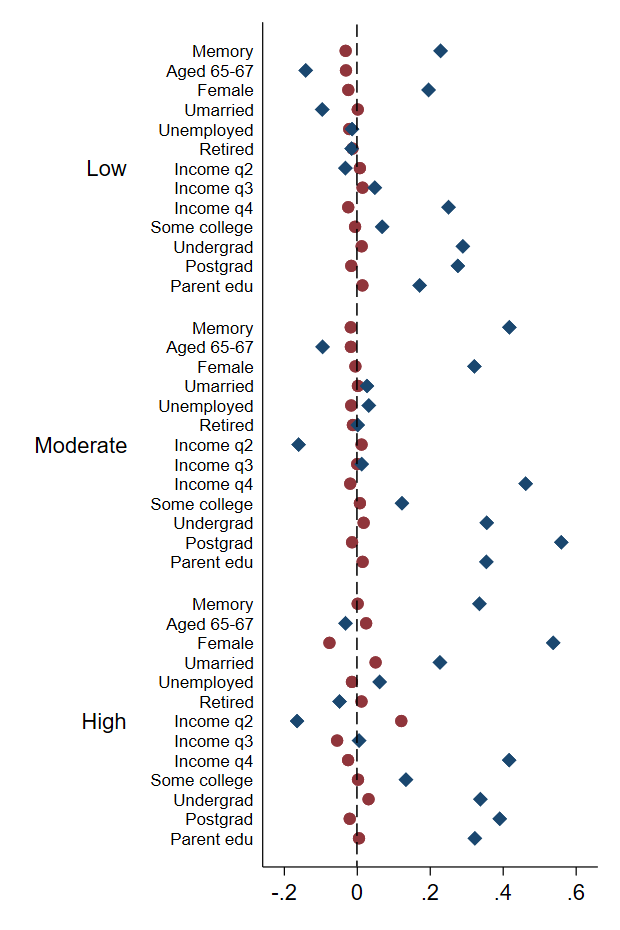


**B**

**A**


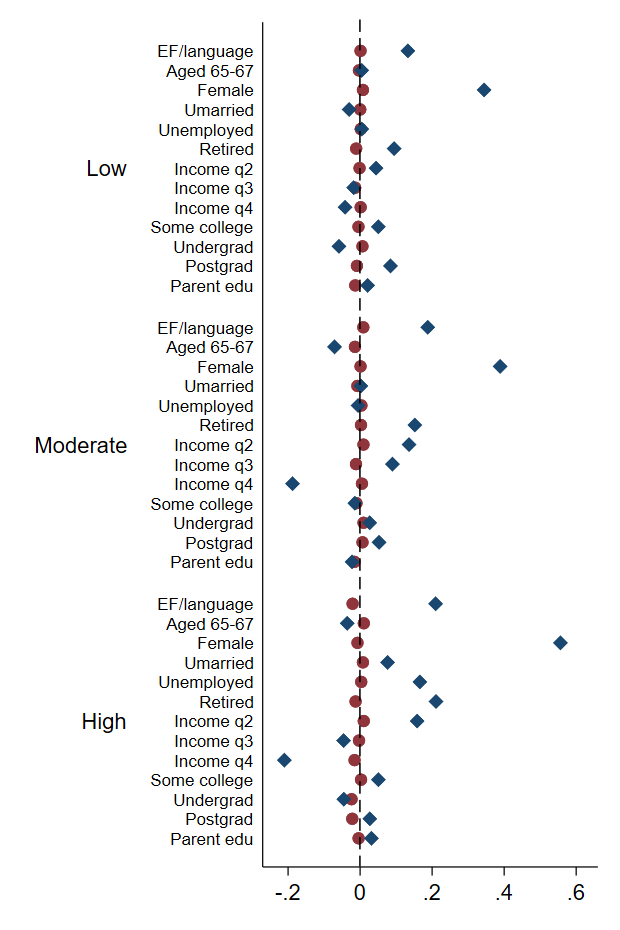

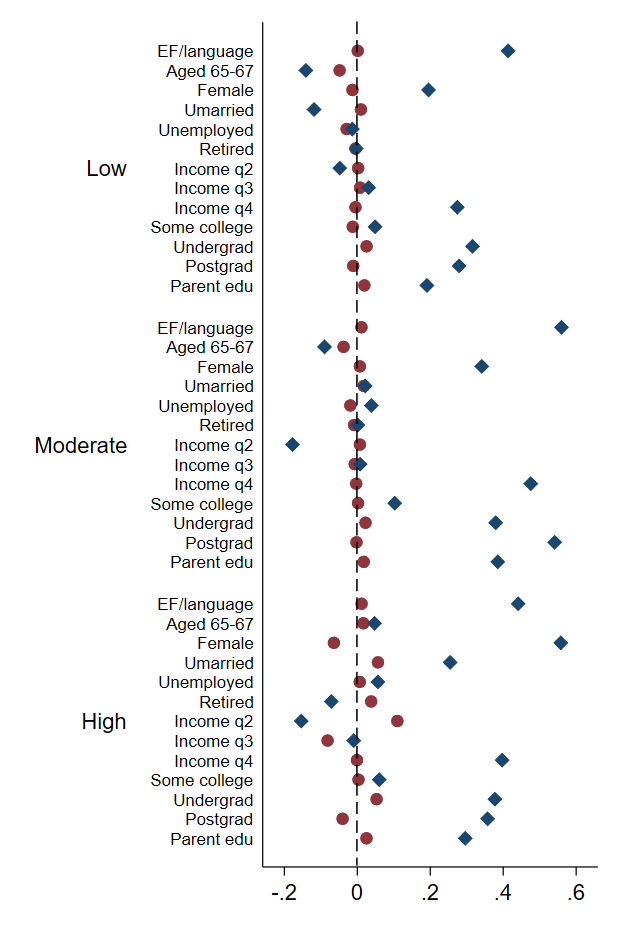


**D**

**C**

*Figure S1.* Covariate balance over treatment groups before (blue diamonds) and after (red circles) inverse probability of treatment weighting. Balance is shown by the standardized difference between each level of arts engagement (low, moderate, high) compared to no arts engagement. A) Participatory arts engagement and memory, N=3,245. B) Receptive arts engagement and memory, N=3,245. C) Participatory arts engagement and EF/language, N=2,926. D) Receptive arts engagement and EF/language, N=2,926.

**Table S1.** *Inverse probability of treatment weighting (IPTW) diagnostics; range and average of standardized differences and variance ratios before and after weighting.*

| **Model** | **Absolute standardized difference** | | | | | | **Variance ratio** | | | | | |
| --- | --- | --- | --- | --- | --- | --- | --- | --- | --- | --- | --- | --- |
|  | **Raw** | | | **Weighted** | | | **Raw** | | | **Weighted** | | |
|  | Min | Max | Avg | Min | Max | Avg | Min | Max | Avg | Min | Max | Avg |
| Participatory arts and memory | 0.01 | 0.53 | 0.06 | 0.00 | 0.02 | 0.00 | 0.81 | 1.39 | 1.02 | 0.94 | 1.06 | 1.00 |
| Receptive arts and memory | 0.00 | 0.56 | 0.16 | 0.00 | 0.12 | 0.00 | 0.83 | 2.82 | 1.32 | 0.92 | 1.12 | 1.00 |
| Participatory arts and EF/language | 0.00 | 0.56 | 0.06 | 0.00 | 0.02 | 0.00 | 0.80 | 1.49 | 1.03 | 0.94 | 1.04 | 1.00 |
| Receptive arts and EF/language | 0.00 | 0.56 | 0.17 | 0.00 | 0.11 | 0.00 | 0.82 | 2.70 | 1.32 | 0.84 | 1.11 | 1.00 |

# Sample characteristics

**Table S2.** *Characteristics of included and excluded participants.*

| **Measure** | **Excluded**  (N=1,376) | **Included:**  **Memory**  (N=3,245) | **Included:**  **EF/language**  (N=2,926) | **Included:**  **Imputation**  (N=4,344) |
| --- | --- | --- | --- | --- |
| Participatory arts engagement |  |  |  |  |
| None | 37% | 39% | 38% | 39% |
| Low | 20% | 21% | 21% | 21% |
| Moderate | 17% | 18% | 19% | 18% |
| High | 26% | 22% | 22% | 22% |
| Receptive arts engagement |  |  |  |  |
| None | 26% | 29% | 29% | 29% |
| Low | 43% | 41% | 41% | 40% |
| Moderate | 27% | 24% | 24% | 25% |
| High | 5% | 6% | 6% | 6% |
| Age: 63-64 years | 61% | 67% | 68% | 65% |
| Gender: men | 46% | 47% | 47% | 47% |
| Marital status: married | 80% | 79% | 79% | 79% |
| Highest education |  |  |  |  |
| High school or less | 54% | 52% | 52% | 54% |
| Some college | 17% | 15% | 15% | 16% |
| Undergraduate | 15% | 15% | 15% | 14% |
| Postgraduate | 13% | 18% | 18% | 16% |
| Employment status |  |  |  |  |
| Employed | 46% | 47% | 46% | 47% |
| Unemployed | 13% | 10% | 11% | 11% |
| Retired | 41% | 43% | 43% | 42% |
| Household income |  |  |  |  |
| $0-$36,000 | 24% | 20% | 20% | 22% |
| $36,001-$57,000 | 24% | 27% | 27% | 26% |
| $57,001-$91,000 | 26% | 26% | 26% | 25% |
| $91,001+ | 27% | 27% | 27% | 27% |
| Parental education: high school or less | 86% | 85% | 85% | 85% |

*Note.* Results in ‘Included: Imputation’ column are based on 20 multiply imputed data sets. Results in the ‘Included: Memory’ and ‘Included: EF/language’ are also shown in Table 1 of the main text.

# Physical activity

**Table S3.** *Associations between physical activity and the two cognition outcomes using inverse probability of treatment weighting.*

|  | **Memory** (n=3,245) | | | **EF/language** (n=2,926) | | |
| --- | --- | --- | --- | --- | --- | --- |
|  | N (%) | ATE | 95% CI | N (%) | ATE | 95% CI |
| None | 1711 (53%) | - | - | 1530 (52%) | - | - |
| Low | 336 (10%) | **0.13** | **0.03 to 0.24** | 303 (10%) | **0.12** | **0.03 to 0.22** |
| Moderate | 524 (16%) | 0.03 | -0.05 to 0.12 | 473 (16%) | 0.07 | -0.01 to 0.15 |
| High | 674 (21%) | -0.01 | -0.10 to 0.07 | 624 (21%) | **0.08** | **0.01 to 0.16** |

*Note.* ATE: average treatment effect. The control group (reference group) was no engagement. Memory and EF/language were standardized, so ATEs are in standard deviation units.

# Inclusion of movies in receptive arts engagement

**Table S4.** *Associations between receptive arts engagement (excluding the movies) and the two cognition outcomes using inverse probability of treatment weighting.*

|  | **Memory** (n=3,245) | | | **EF/language** (n=2,926) | | |
| --- | --- | --- | --- | --- | --- | --- |
|  | N (%) | ATE | 95% CI | N (%) | ATE | 95% CI |
| None | 1373 (38% | - | - | 1237 (38%) | - | - |
| Low | 1717 (48%) | **0.11** | **0.04 to 0.19** | 1552 (48%) | **0.12** | **0.06 to 0.19** |
| Moderate | 424 (12%) | 0.02 | -0.07 to 0.15 | 390 (12%) | 0.00 | -0.14 to 0.14 |
| High | 57 (2%) | -0.17 | -0.58 to 0.24 | 50 (2%) | 0.19 | -0.12 to 0.51 |

*Note.* ATE: average treatment effect in standard deviation units. The control group was no engagement.

**Table S5.** *Associations between going to the movies (excluding other forms of receptive arts engagement) and the two cognition outcomes using inverse probability of treatment weighting.*

|  | **Memory** (n=3,245) | | | **EF/language** (n=2,926) | | |
| --- | --- | --- | --- | --- | --- | --- |
|  | N (%) | ATE | 95% CI | N (%) | ATE | 95% CI |
| None | 1769 (49%) | **-** | **-** | 1579 (49%) |  |  |
| Low | 1383 (38%) | **0.09** | **0.02 to 0.16** | 1248 (39%) | 0.03 | -0.03 to 0.09 |
| Moderate | 394 (11%) | 0.06 | -0.05 to 0.17 | 371 (11%) | -0.03 | -0.14 to 0.07 |
| High | 50 (1%) | -0.03 | -0.24 to 0.17 | 43 (1%) | -0.16 | -0.46 to 0.13 |

*Note.* ATE: average treatment effect in standard deviation units. The control group was no engagement.

# Binary indicators of arts engagement

**Table S6.** *Associations between a binary indicator of arts engagement (none vs engaged) and the two cognition outcomes using inverse probability of treatment weighting.*

|  | **Memory** (n=3,245) | | | | | | **EF/language** (n=2,926) | | | | | |
| --- | --- | --- | --- | --- | --- | --- | --- | --- | --- | --- | --- | --- |
|  | **Participatory arts** | | | **Receptive arts** | | | **Participatory arts** | | | **Receptive arts** | | |
|  | % | ATE | 95% CI | % | ATE | 95% CI | % | ATE | 95% CI | % | ATE | 95% CI |
| Engaged | 61% | 0.03 | -0.03 to 0.10 | 71% | **0.08** | **0.01 to 0.16** | 62% | **0.06** | **0.00 to 0.12** | 71% | 0.07 | -0.01 to 0.14 |

*Note.* ATE: average treatment effect in standard deviation units. For both participatory and receptive arts engagement, the control group was no engagement.

# Regression models

**Table S7.** *Associations between arts engagement and the two cognition outcomes using linear regression models.*

|  | **Memory** (n=3,245) | | | | **EF/language** (n=2,926) | | | |
| --- | --- | --- | --- | --- | --- | --- | --- | --- |
|  | **Unadjusted** | | **Adjusted** | | **Unadjusted** | | **Adjusted** | |
|  | Coef (95% CI) | p value | Coef (95% CI) | p value | Coef (95% CI) | p value | Coef (95% CI) | p value |
| **Participatory arts engagement** | | | | | | | | |
| Low | 0.06 (-0.02 to 0.15) | 0.154 | 0.02 (-0.07 to 0.10) | 0.692 | **0.09 (0.01 to 0.16)** | **0.026** | **0.08 (0.00 to 0.15)** | **0.040** |
| Moderate | **0.11 (0.02 to 0.20)** | **0.016** | 0.06 (-0.03 to 0.15) | 0.186 | 0.08 (-0.00 to 0.15) | 0.054 | 0.07 (-0.01 to 0.14) | 0.087 |
| High | 0.05 (-0.03 to 0.14) | 0.215 | 0.00 (-0.08 to 0.09) | 0.975 | 0.05 (-0.02 to 0.12) | 0.189 | 0.06 (-0.02 to 0.13) | 0.139 |
| **Receptive arts engagement** | | | | | | | | |
| Low | **0.20 (0.12 to 0.27)** | **<0.001** | **0.11 (0.04 to 0.19)** | **0.004** | **0.17 (0.10 to 0.24)** | **<0.001** | **0.10 (0.03 to 0.17)** | **0.004** |
| Moderate | **0.23 (0.14 to 0.31)** | **<0.001** | **0.09 (0.00 to 0.18)** | **0.049** | **0.15 (0.07 to 0.22)** | **<0.001** | 0.02 (-0.06 to 0.10) | 0.661 |
| High | 0.12 (-0.02 to 0.26) | 0.101 | -0.01 (-0.16 to 0.13) | 0.855 | **0.19 (0.06 to 0.31)** | **0.003** | 0.08 (-0.05 to 0.20) | 0.222 |

*Note.* Participatory and receptive arts engagement were included as exposures in separate models. For both, the reference category was no engagement. Adjusted models included gender, age, marital status, education, employment status, household income, and parental education. All models adjusted for baseline cognition. Coefficients in standard deviation units. Bold text indicates p<0.05.

# Imputed analyses

**Table S8.** *Proportion of missing data in the imputation sample.*

| **Measure** | **Missing** |
| --- | --- |
| Participatory arts engagement | 18% |
| Receptive arts engagement | 16% |
| Physical activity | 17% |
| Age | 0 |
| Gender | 0 |
| Marital status | 0 |
| Highest education | 0.02% |
| Employment status | 0 |
| Household income | 0 |
| Parental education | 1% |
| General health rating | 0.02% |
| Memory (baseline) | 2% |
| Memory (follow-up) | 2% |
| EF/language (baseline) | 8% |
| EF/language (follow-up) | 7% |

Note. n=4,344. General health rating was an auxiliary variable in the imputation model and was not included in subsequent analyses.


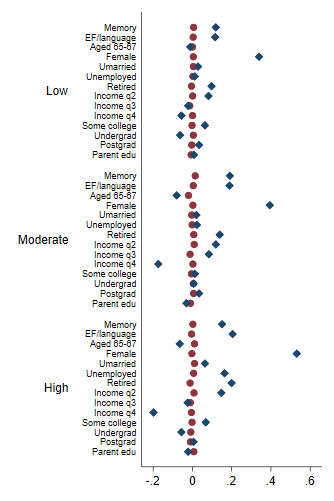

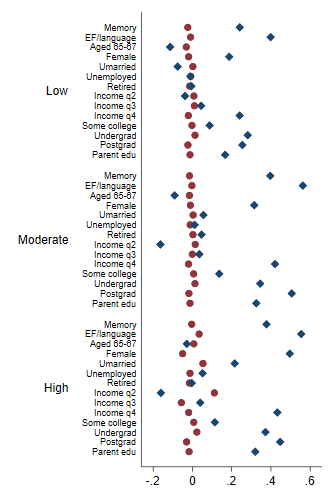


**B**

**A**

*Figure S2.* Covariate balance over treatment groups before (blue diamonds) and after (red circles) inverse probability of treatment weighting. Balance is shown by the standardized difference between each level of arts engagement (low, moderate, high) compared to no arts engagement. N=4,344. A) Participatory arts engagement. B) Receptive arts engagement.

**Table S9.** *Associations between arts engagement and the two cognition outcomes using inverse probability of treatment weighting combined with multiple imputation using the “conventional approach”.*

|  | **Memory** | | | | | | **EF/language** | | | | | |
| --- | --- | --- | --- | --- | --- | --- | --- | --- | --- | --- | --- | --- |
|  | **Participatory arts** | | **Receptive arts** | | **Physical activity** | | **Participatory arts** | | **Receptive arts** | | **Physical activity** | |
|  | ATE | 95% CI | ATE | 95% CI | ATE | 95% CI | ATE | 95% CI | ATE | 95% CI | ATE | 95% CI |
| Low | 0.04 | -0.04 to 0.12 | **0.10** | **0.03 to 0.18** | **0.13** | **0.03 to 0.23** | 0.07 | -0.01 to 0.14 | **0.10** | **0.03 to 0.18** | **0.12** | **0.03 to 0.20** |
| Moderate | **0.09** | **0.00 to 0.17** | **0.14** | **0.04 to 0.23** | 0.05 | -0.04 to 0.14 | **0.09** | **0.00 to 0.17** | **0.11** | **0.03 to 0.19** | **0.10** | **0.02 to 0.18** |
| High | 0.03 | -0.05 to 0.12 | 0.08 | -0.06 to 0.22 | 0.03 | -0.05 to 0.11 | 0.07 | -0.01 to 0.15 | 0.12 | -0.03 to 0.27 | **0.13** | **0.05 to 0.21** |

*Note.* N=4,344. After imputation, IPTW was performed using *“teffects ipw”* and *“mi estimate”* Stata commands.

**Table S10.** *Associations between arts engagement and the two cognition outcomes using inverse probability of treatment weighting combined with multiple imputation using the “within approach”.*

|  | **Memory** | | | | | | **EF/language** | | | | | |
| --- | --- | --- | --- | --- | --- | --- | --- | --- | --- | --- | --- | --- |
|  | **Participatory arts** | | **Receptive arts** | | **Physical activity** | | **Participatory arts** | | **Receptive arts** | | **Physical activity** | |
|  | ATE | 95% CI | ATE | 95% CI | ATE | 95% CI | ATE | 95% CI | ATE | 95% CI | ATE | 95% CI |
| Low | 0.03 | -0.35 to 0.41 | 0.08 | -0.30 to 0.45 | 0.10 | -0.33 to 0.53 | 0.06 | -0.29 to 0.41 | 0.05 | -0.30 to 0.40 | 0.09 | -0.31 to 0.48 |
| Moderate | 0.07 | -0.32 to 0.46 | 0.08 | -0.33 to 0.48 | 0.02 | -0.37 to 0.40 | 0.08 | -0.28 to 0.44 | 0.02 | -0.36 to 0.40 | 0.07 | -0.28 to 0.43 |
| High | 0.02 | -0.37 to 0.41 | 0.01 | -0.50 to 0.52 | -0.02 | -0.39 to 0.36 | 0.07 | -0.29 to 0.43 | 0.03 | -0.47 to 0.53 | 0.09 | -0.26 to 0.44 |

*Note.* N=4,344. After imputation, IPTW was performed individually in each imputation to obtain 20 effect estimates. These estimates were then combined using Rubin’s rules to produce estimates of overall exposure effects and confidence intervals (with standard errors comprised of both the between-imputation and within-imputation variance).
